# Highly versatile small virus-encoded proteins in cellular membranes: A structural perspective on how proteins’ inherent conformational plasticity couples with host membranes’ properties to control cellular processes^☆^

**DOI:** 10.1016/j.yjsbx.2024.100117

**Published:** 2024-12-11

**Authors:** Arvin Saffarian Delkhosh, Elaheh Hadadianpour, Md Majharul Islam, Elka R. Georgieva

**Affiliations:** Department of Chemistry and Biochemistry, Texas Tech University, Lubbock, TX 79409, USA

**Keywords:** Viral membrane proteins, Viroporins, Protein structure, Protein conformational dynamics, Viral protein-induced membrane permeability to ions

## Abstract

We investigated several small viral proteins that reside and function in cellular membranes. These proteins belong to the viroporin family because they assemble into ion-conducting oligomers. However, despite forming similar oligomeric structures with analogous functions, these proteins have diverse amino acid sequences. In particular, the amino acid compositions of the proposed channel-forming transmembrane (TM) helices are vastly different—some contain residues (e.g., His, Trp, Asp, Ser) that could facilitate cation transport. Still, other viroporins’ TM helices encompass exclusively hydrophobic residues; therefore, it is difficult to explain their channels’ activity, unless other mechanisms (e.g., involving a negative lipid headgroups and/or membrane destabilization) take place. For this study, we selected the M2, Vpu, E, p13II, p7, and 2B proteins from the influenza A, HIV-1, human T-cell leukemia, hepatitis C, and picorna viruses, respectively. We provide a brief overview of the current knowledge about these proteins’ structures as well as remaining questions about more comprehensive understanding of their structures, conformational dynamics, and function. Finally, we outline strategies to utilize a multi-prong structural and computational approach to overcome current deficiencies in the knowledge about these proteins.

## Highlights

• Small virus-encoded proteins homo-oligomerize in cellular membranes and function as ion channels.
• These proteins were combined in the family of viroporins.
• Despite the similarity in their oligomeric structures and functions, these proteins have vastly different primary structures.
• It is imperative to understand how proteins with no homology in their primary structures fulfill similar functions for diverse viruses.
• A multidisciplinary approach has the potential to explain the structure, conformational dynamics, and function of these proteins.

## Graphical abstract

**Figure:**
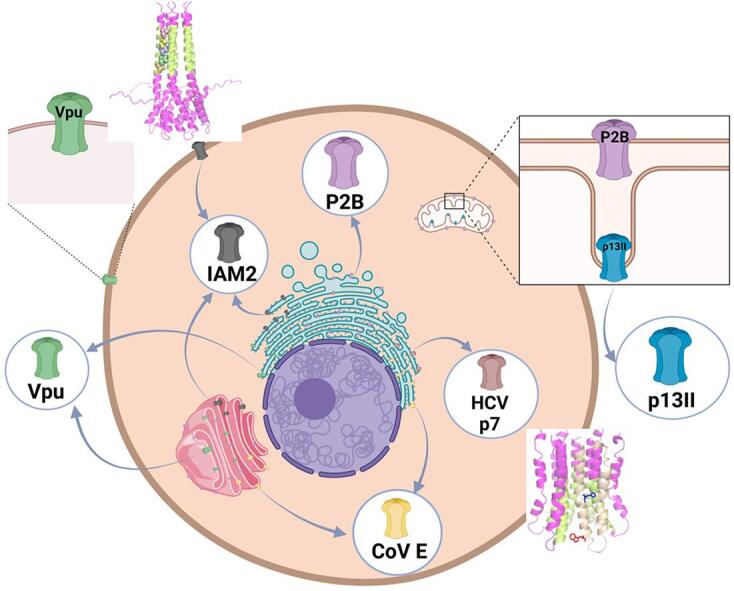

## Introduction

Pathogenic viruses are among the key environmental factors with a profound negative effect on human health and the sustainable development of human society (https://www.un.org/sustainabledevelopment/). Understanding their mechanisms in hosts is critical for the prevention and overcoming of viral infections. To this end, it is imperative to understand the structure–function relationship of key viral proteins, which will be informative for the design of effective anti-viral therapeutics.

**Fig. 1:**
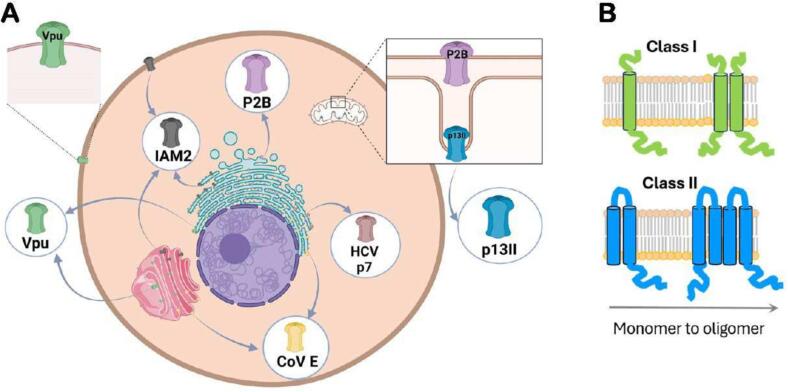
**Small viral proteins (viroporins) in host’s cell membranes.** (A) These proteins reside and function in plasma and organelle membranes (e.g., membranes of ER and Golgi, and mitochondrial membranes). Furthermore, some of them are found in more than one membrane, e.g., IAM2 and Vpu are located in both plasma and endomembranes (ER and Golgi); P2B is found in endomembranes and outer mitochondrial membrane (OMM). The figure was created in BioRender. (B) The Classes I (1-TM helix) and II (2-TM helices) viroporins are shown; homo-oligomers are formed via TM helix-TM helix associations (only two protomers are shown for clarity).

The focus here is on small viral proteins that reside and function in the cellular membranes of infected cells (Fig. 1 A). They consist of 50–120 amino acid residues with one or two transmembrane (TM) helices. These proteins are collectively called viroporins because of their ability to form oligomers with ion-conducting channel or pore activities; they also play a role in virus budding.(Seelamgari et al., 2004, Malim and Emerman, 2008, Fischer and Hsu, 2011, DiMaio, 2014, Luis Nieva and Carrasco, 2015, Scott and Griffin, 2015, Manzoor et al., 2017, Georgieva, 2018, Raheja et al., 2023) The influenza A M2 (IAM2) protein was the first viroporin identified and characterized.(Lamb et al., 1985, Sugrue and Hay, 1991, Pielak and Chou, 2011) After that, viroporins’ family expanded significantly, and such small proteins encoded by most currently known viruses have been identified. Besides IAM2, prominent examples of viroporins include the influenza B M2 (IBM2),(Imai, Watanabe et al., 2004) HIV-1 Vpu,(Strebel et al., 1988, Gonzalez, 2015) coronavirus (CoV) E,(McClenaghan et al., 2020, Santos-Mendoza, 2023) hepatitis C virus (HCV) p7,(Chandler et al., 2012, Atoom et al., 2014) and picornavirus 2B (P2B) proteins,(de Jong, de Mattia et al., 2008) as well as human T-cell leukemia virus type I (HTLV-1) p13II protein.(Silic-Benussi et al., 2010, Georgieva, 2018) Commonly, viroporins are subdivided into two classes (Fig. 1 B). The Class I consists of members with one TM helix (e.g., IAM2, IBM2, HIV-1 Vpu, CoV E); viroporins with two membrane-traversing helices (e.g., HCV p7, P2B) belong to Class II.(Nieva, Madan et al., 2012) The TM helices of several monomers (the number of monomers is protein-specific) interact in the membrane to form homo-oligomeric structures, which increase membrane permeability for ions.

In addition to their capacity to increase membrane permeability, these proteins participate in diverse protein–protein interactions, forming hetero-protein complexes via their TM helices or soluble domains. Through these interactions, they redirect host signaling pathways by antagonizing host proteins and aiding virus budding.(Seelamgari et al., 2004, Malim and Emerman, 2008, Zhu et al., 2016, Georgieva, 2018, Raheja et al., 2023) Therefore, these proteins are highly functionally versatile and useful for the respective viruses.

## A brief description of the current status of viroporins’ structural elucidations

**Fig. 2:**
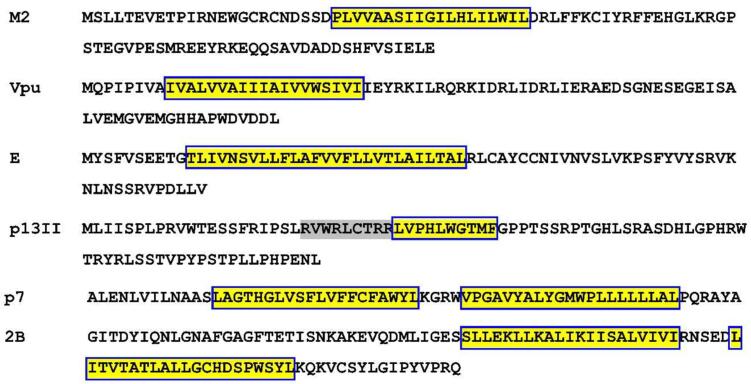
**The amino acid sequences of the IAM2 (accession# P63231.1), HIV-1 Vpu (accession#NP_057855.1), CoV E (accession#YP_009724392.1), HTCLV-1 p13II (accession#AAB23362.1), HCV p7 (accession# YP-009709864.1, and P2B (accession# YP-009508946.1) are shown.** The TM helices are highlighted in yellow and boxed. The region with positively charged residues in p13II’s sequence is highlighted in gray. The proteins were randomly selected aiming to demonstrate the distinct amino acid composition, particularly the composition of the TM helices, which associate to form ion-conducting channels or pores. (For interpretation of the references to colour in this figure legend, the reader is referred to the web version of this article.). (For interpretation of the references to colour in this figure legend, the reader is referred to the web version of this article.)

**Fig. 3:**
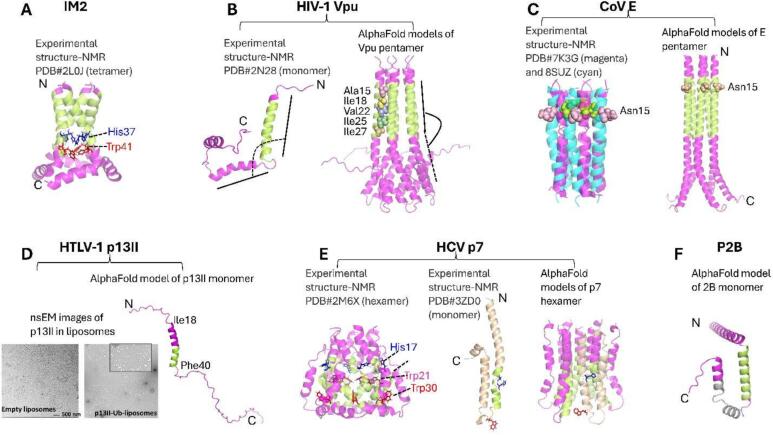
**The experimental and predicted structures of IAM2, HIV1 Vpu, CoV E, HICV p7, P2B and HTLV1 p13II proteins.** (A) The NMR structure of IAM2 tetramer is shown. The TM helices are in green. The His37 and Thr41 critical for proton’s translocation are shown as sticks in blue and red, respectively. (B) The NMR structure of Vpu monomer (left) and AlphaFold-predicted structure of Vpu pentamer (right) are shown. The TM helices are in green. Based on the NMR structure, helix 2 (after TM helix) is bound to the membrane surface making almost 90° angle with TM helix 1; whereas, based on the AlphaFold prediction, helix 2 is almost a straight continuation of TM helix 1 outside of the membrane. These might correspond to different Vpu conformations, but further clarification would be necessary. Strikingly, all residues in TM helix facing the pentamer interior are highly hydrophobic, and how the channel/pore might transport ions is currently unclear. (C) The NMR structures of pentameric E protein TM domain in closed and open conformations are overlayed (left). The experimental results suggest that local restructuring, including helix rotation, at and around residue Asn15 leads to channel/pore opening to facilitate ion translocation. The AlphaFold predicted structure of E protein pentamer is shown. In this model, even in closed state, the Asn15 residue does not point to the inward of the pore. (D) nsEM data of p13II protein reconstituted in DOPC/DOPS liposomes at 1:300 protein-to-lipid molar ratio is shown (left)—The protein reconstitution and nsEM were conducted as described in Georgieva *at al*, *Prot. Expr. Purif.*, 2020 and Majeed *et al*, *J. Struct. Biol*, 2023, respectively (cf. Supplementary Information). Large protein clusters were observed. The AlphaFold model of p13II monomer is shown. The predicted TM helix is in green and the amphipathic helix containing several Arg residues is in magenta. (E) The NMR structure of p7 hexamer is shown (left)— unusual fold was observed, the channel is formed with participation of TM helices of each protomer. Another NMR structure of p7 monomer is shown, which is substantially different from the monomer structure in the assembled hexamer (left). This could be because these are the structures of p7 from different genotypes. The AlphaFold model of p7 hexamer is shown on the right. The monomer structure in this oligomer is closer to the structure in the middle. Two conserved residues His17 and Trp30 were identified as possibly participating in ion translocation, but further studies to confirm this are necessary. (F) The AlphaFold model of 2B protein is shown. The predicted TM helices are in green and gray, respectively. All AlphaFold structure predictions were run from ChimeraX (cf. Supplementary Information). (For interpretation of the references to colour in this figure legend, the reader is referred to the web version of this article.). (For interpretation of the references to colour in this figure legend, the reader is referred to the web version of this article.)

The structures of several viroporins in varying oligomeric states have been characterized to certain extents. However, the structures of most of the proposed viroporins have not been solved at all. Moreover, the differences in oligomerization profiles of Class I and Class II virporins are unknown. Here, we focus on proteins encoded by six diverse representative viruses—the IAM2, HIV-1 Vpu, CoV E, HCV p7, P2B, and HTLV-1 p13II. The amino acid sequences of these proteins and their TM helices are shown in Fig. 2. The experimentally determined structures for some of these proteins and/or those generated in this study structures’ predictions using the AlphaFold multimer program(Bryant et al., 2022, Wallner, 2023) are shown in Fig. 3.

IAM2 has been a paradigm in studies of channel-/pore-forming homo-oligomeric viral proteins. The protein assembles via its TM helix in a proton-specific tetrameric channel.(Pielak and Chou, 2011, Manzoor et al., 2017) Multiple tetrameric structures of full-length (FL) IAM2 and its truncated constructs containing the TM helix were reported by NMR and X-ray crystallography under various lipid, detergent, and pH conditions and supported by MD simulations.(Zhong et al., 1998, Duong-Ly et al., 2005, Cady et al., 2007, Hu et al., 2007, Schnell and Chou, 2008) It was determined that IAM2′s residues H37 and W41 play the major role in proton translocation across the membrane;(Hu et al., 2006, Acharya et al., 2010). However, no role of these residues in protein oligomerization has been identified. It was found in an earlier continuous wave ESR (CW ESR) study that the lipid bilayer thickness affects the conformation of IAM2, as the protein tetramer formation and rigidity were more pronounced in lipid bilayers matching the thickness of IAM2 transmembrane helix.(Duong-Ly, Nanda et al., 2005) This is in agreement with a later study using pulse ESR (DEER) spectroscopy.(Georgieva, Borbat et al., 2016) Through DEER spectroscopy study of IAM2 in model liposomes, it was found that IAM2′s tetramer assembles via a tandem mechanism, which is a monomer-to-a dimer-to-a dimer of dimers (tetramer).(Georgieva, Borbat et al., 2015) This mechanism was confirmed for IAM2 reconstituted in isolated *E. coli* membranes containing native lipids and proteins under very close-to-native membrane conditions.(Sanders, Borbat et al., 2024) Additionally, ESR studies shed light on the dynamics of the membrane-bound helix 2 (the IM2 region following the TM helix) of IM2.(Huang, Green et al., 2015) The role of cholesterol in shaping the IAM2 tetramer was also proposed and computational studies confirmed that cholesterol affects the clustering of IAM2′s tetramers.(Ekanayake et al., 2016, Elkins et al., 2017, Kolokouris et al., 2024).

NMR spectroscopy and X-ray crystallography provided insights into the structure of HIV-1 Vpu protein’s isolated C-terminal region,(Lu et al., 2010, Jia et al., 2014) TM helix,(Lu, Sharpe et al., 2010) and FL Vpu monomer.(Ma, Marassi et al., 2002) Based on these studies, a pentameric Vpu’s organization in the membrane was proposed and confirmed by computational studies.(Grice, Kerr et al., 1997) To explain the ion conductivity of Vpu oligomer, it was proposed that a kink in the transmembrane helix at residue Ile17 might create conditions for the translocation of cations.(Park, Mrse et al., 2003) In addition, MD simulations of Vpu’s oligomer in membrane suggested that the residue Ser23 points toward the putative oligomeric pore,(Grice, Kerr et al., 1997) which might be relevant to ion translocation. Recently, we found that Vpu can self-associate outside of lipid environments, forming predominantly stable hexamers.(Majeed et al., 2023, Majeed et al., 2023) However, further investigations would be needed to understand whether Vpu’s soluble and membrane-bound oligomers share similarities.

In lipids, CoV’s E protein oligomerizes through its TM helix, possibly in a homopentamer with ion-channel activity.(Torres et al., 2007, Nieto-Torres et al., 2014, Surya et al., 2018, Schoeman and Fielding, 2019, Mandala et al., 2020) A study using NMR and MD simulations found that the structure of CoV E pentamer is relatively rigid, compared to the more flexible structure of IAM2 tetramer, and mostly hydrophobic residues face the interior of the putative channel pore.(Mandala, McKay et al., 2020) Further computational modeling predicted that the hydrophobic domain (helix) of CoV E could form stable dimers, trimers, and pentamers.(Torres, Wang et al., 2005) An experimentally observed dimeric structure of membrane-bound E protein was reported as well.(Zhang, Qin et al., 2023) Recent MD simulations of the pentameric CoV E in lipid suggested that despite the amino acid residues contributing to the channel being mostly hydrophobic (except residues Glu8 and Asn15) the channel was well hydrated due to water molecules penetration the lipid bilayer,(Yang, Wu et al., 2022) which might help to explain the ion conductivity of this protein. Interestingly, a recent study found that in lipid bilayers, the protein can form clusters of pentamers,(Somberg, Wu et al., 2022) similar to the observed IAM2 clusters.(Sutherland, Tran et al., 2022).

The HCV p7 protein localizes primarily on the ER’s membrane with both termini, N- and C-, facing toward the ER lumen,(Khaliq, Jahan et al., 2011) but it also targets mitochondrial membrane.(You, Lee et al., 2017) It belongs to the Class II viroporins, as it has two TM helices connected by a short cytoplasmic loop.(Atoom, Taylor et al., 2014) In membranes, p7 forms cation-conducting oligomers (channel or pore).(Chandler et al., 2012, Atoom et al., 2014, You et al., 2017) It is known from NMR and single-particle electron microscopy (EM) that p7 forms a hexameric unit, with each protomer contributing to ion translocation.(Luik et al., 2009, Madan and Bartenschlager, 2015) However, heptameric(Clarke, Griffin et al., 2006) and pentameric(Pavlovic, Neville et al., 2003) forms of this protein were also reported. Therefore, p7′s oligomerization profile is not well understood, similarly to Vpu and E proteins.

Despite these advances in elucidating the structures and conformational plasticity of several members of the viroporin family, the structures of many of these proteins have not been determined. One such protein is P2B, which has an important role in the life cycle of picornaviruses.(Li, Zou et al., 2019) The structural studies of this protein have not been adequately advanced. It is known from amino acid sequence analysis that this protein has two putative TM helices, TM1 and TM2, arranged in α-helix-turn-α-helix motif.(Li, Zou et al., 2019) It is proposed that the protein assembles in a tetramer,(Agirre et al., 2002, Patargias et al., 2009, Li et al., 2019) the structure of which is currently uncharacterized.

Another interesting protein classified as a viroporin is the HTLV-1 encoded p13II protein,(Silic-Benussi et al., 2010, Scott and Griffin, 2015) which associates with the inner mitochondrial membrane (IMM), where it forms a cation-conducting oligomeric channel.(Silic-Benussi et al., 2009, Biasiotto et al., 2010, Silic-Benussi et al., 2010, Silic-Benussi et al., 2010) However, very little is known about the structure of this protein in the IMM. The formation of oligomers capable of depolarizing IMM has been reported.(Silic-Benussi, Biasiotto et al., 2010, Silic-Benussi et al., 2010) In a recent study, the production of large quantities of highly-pure p13II was reported and found using ESR spectroscopy that this protein oligomerized in lyso lipid and lipid bilayers; the protein also induced Tl^+^ permeability of liposomes, as inferred by fluorescence spectroscopy.(Georgieva, Borbat et al., 2020) Recently, we used negative staining EM (nsEM) to observe large liposome-bound p13II clusters (Fig. 3D), which might indicate that membrane permeability is achieved via a different mechanism than those of a channel or pore. Further studies would be needed to characterize these p13II clusters, including solving the structures of the p13II monomer and oligomer.

Thus, in spite of their critical roles in virus adaptation and proliferation, studies on the structure and conformational dynamics of these small membrane-residing viral proteins are incomplete.

## Persisting questions in studies of viroporins

It is currently unknown how viroporins respond structurally to interactions with cellular components (e.g., cellular membranes and host or other viral proteins). It is plausible that these proteins possess high conformational plasticity and can adopt multiple tertiary and quaternary structures to fulfill their diverse functions. However, the small size of these proteins and their membrane localization makes it challenging to assess and characterize each of these structures and to capture the conformational dynamics underlying the transitions between proteins’ functional states. It would be indeed captivating to characterize in detail the varying conformations of these proteins and identify the factors controlling the isomerization between them. In particular, we are interested to understand how viroporins assemble into oligomers to facilitate ion transport across the membrane, and what is the nature of these oligomers. One staggering question is whether membrane’s permeability to ions increases as a result of the activity of one particular protein’s oligomeric state and whether large proteins’ clusters, such as those observed for IAM2,(Sutherland, Tran et al., 2022) CoV E,(Somberg, Wu et al., 2022) and HTLV1 p13II (Fig. 2D), are needed for membrane destabilization. Additionally, there is no clear understanding as to whether these viroporins can form complexes with other protein partners (e.g., host or viral proteins), preserving their channel/pore conformation or the homo-oligomers (and monomers) need to restructure. Hence, key aspects of the structure–function relationship of these proteins require further elucidation.

Below, we summarize our view on three major persisting questions about channel/pore-forming small viral proteins.

### How do proteins with no homology in their amino acid sequences form similar homo-oligomeric structures in the host cellular membranes to fulfill analogous functions?

Careful examination of the primary structures of the proteins from the viroporin family shows no homology among them (Fig. 2). Therefore, it is not clear whether these proteins form a family at all, as their amino acid sequences have little in common, and no evolutionary link is known. It may well be that to gain efficiency, the viruses evolved independently from each other to encode small membrane-residing proteins with similar properties (at least in their capacity to form oligomers in the membrane, thus increasing membrane permeability for ions). A possible approach to put together this fragment of the viroporins’ puzzle is to conduct parallel structural/conformational dynamics studies on several viroporins. This might help to juxtapose the oligomerization mechanisms of a set of proteins, thus uncovering the similarities in their behavior but also singling out the individual properties of each protein.

We believe that oligomerization is an intrinsic property of these small viral membrane proteins, having been observed experimentally for many of the family members. Still, further studies would be needed to link their particular oligomeric structures to specific functions.

### How do the amino acid sequences of these proteins couple with the membrane properties to assemble into distinct structures fulfilling specific functions?

Strikingly, unlike the host DNA-encoded TM proteins, which reside and functions in specific membranes, each of the functional viroporins is typically found in different environments (e.g., the plasma membrane, ER and Golgi membranes, and OMM and IMM).(Hussain et al., 2007, Silic-Benussi et al., 2010, Nieto-Torres et al., 2011, Gonzalez, 2015, Manzoor et al., 2017) This suggests proteins’ high adaptability, as they can adjust to a variety of lipid environments (lipid composition, membrane thickness, charge, cholesterol content, etc.), seemingly an advantageous property most likely predefined by their primary structures. Moreover, some of these proteins were found in oligomeric and monomeric forms in different organelle membranes—HIV-1 Vpu protein forms a homopentamer in the membranes of Golgi and intracellular vesicles, but not in the ER’s membrane,(Hussain, Das et al., 2007) confirming that the functional Vpu can transition between multiple quaternary structures.

It is now well understood that the functional oligomeric order of TM proteins is controlled by the helix–helix and helix–lipid interactions (driven by Van der Waals forces, hydrogen bonding, and electrostatic and aromatic interactions).(Cymer et al., 2012, Li et al., 2012, Stangl and Schneider, 2015) The 3D structure of these proteins is largely determined by their amino acid sequences, but the membrane hydrophobic thickness, fluidity, and charge, which are results of the finely-tuned lipid makeup, are essential for proper folding and assembly of these proteins.(White and Wimley, 1999, Corin and Bowie, 2022, Levental and Lyman, 2023) In particular, it was found that IAM2 tetramer assembles more efficiently in lipids with longer hydrocarbon chains (DOPC/POPS) vs. lipids with shorter hydrocarbon chains (DLPC/DLPS).(Georgieva, Borbat et al., 2016) The IAM2's structural dependence on the length of the lipid hydrocarbon chain was confirmed for protein residing in DMPC vs. DOPC bilayers.(Kovacs, Denny et al., 2000).

Additional studies by others and in our lab show that under the same conditions, the homo-oligomerization properties of various viroporins (e.g., HIV-1Vpu vs. CoV E)(Majeed et al., 2023, Townsend et al., 2024) are different. This suggests that each of these proteins uses a subtle individual style to couple its amino acid sequence with the lipid environment to fold into a certain quaternary structure (this possibly affects the tertiary structure as well). It is now well understood that the energy landscape and conformational variability, including oligomeric states, of viroporins are wide-ranging; therefore, the detailed characterization of viroporins is challenging.(Devantier, Kjaer et al., 2024) From the currently available information, one could assume that the ion-conducting oligomers are formed under particular membrane conditions, but viroporins’ communication with the other viral or host components (e.g., protein–protein hetero-complexes formation) in the infected cell might also initiate the oligomer formation or dissociation.

### How can the existing experimental and/or predicted structure of viroporins’ oligomers explain the function of these proteins as ion channels or pores?

Several biophysical and structural biology techniques were utilized to elucidate viroporins’ structures. NMR, X-ray crystallography, and nsEM visualized the IAM2 tetramer, Vpu pentamer, E pentamer and p7 hexamer.(Zhong et al., 1998, Ma et al., 2002, Duong-Ly et al., 2005, Schnell and Chou, 2008, Luik et al., 2009, Georgieva et al., 2015, Madan and Bartenschlager, 2015, Mandala et al., 2020) Particularly important for understanding the mechanism of ion conductance are the TM domain structures forming the channel/pore across the membrane. It is now well understood that IAM2 is almost exclusively a proton channel, and the conserved His37 and Trp41 (Fig. 3A) play critical roles in proton translocation.(Pielak and Chou, 2011) A similar arrangement of residue His17 and Trp30 is found in the TM region of the p7 protein (Fig. 3E), but no proton specificity or plausible mechanism of ion translocation have been determined for this protein to date. It has been proposed that the E protein channel opens upon an ion entering the pore, triggering TM helix rotation and restructuring in the region of residues N15 and S16 (Fig. 3C), thus forming a water-filled pore to facilitate the ion translocation. Still, this could explain only halfway of the ion movement in the membrane. The rest of the amino acids in E’s TM are highly hydrophobic and, therefore, may not be suited to provide an ion-translocation pathway. It is even more complex in the case of Vpu—its TM helix’s composition is very hydrophobic (Fig. 3B), and there is currently no known mechanism to explain the ion-conducting mechanism of this pentamer. Transient water wires were proposed to mediate ion movement through patches of hydrophobic amino acids in the TM channel.(Kratochvil, Watkins et al., 2023) However, it is not plausible to imagine a water wire spanning 20 hydrophobic amino acids in the case of Vpu. Another proposed mechanism is the formation of a lipid-protein pore, in which the negatively charged lipid headgroups face the pore.(Nieto-Torres, Verdia-Baguena et al., 2015) However, such a structure has not been visualized to date.

Another challenging case is to explain how p13II can span the IMM, given that the proposed TM helix contains only about 10 amino acids (Fig. 2 and Fig. 3D), which can form a helix with length of about 15 Å, thus insufficient to traverse a membrane with hydrophobic thickness of > 25 Å. Then the question is: Does the amphipathic helix preceding the predicted TM helix contribute to the channel as well, possibly through forming a water-filled pore, which penetrates the membrane? This scenario cannot be ruled out, although it would be difficult to explain the transport of cations, as the p13II’s amphipathic helix preceding the TM helix contains several positively charged arginine residues and might cause cation repulsion. Another possibility is that p13II’s TM helices do not fully traverse the membrane but anchor in the bilayer and collectively produce a membrane defect. The region with high arginine content could also play a role in membrane association through interactions with negative lipid headgroups. However, this is yet to be established.

## The need for a multi-prong approach to elucidate the structures and conformational dynamics of proteins from the viroporin family

The importance of the channel-/pore-forming small viral membrane proteins is undisputable because they fulfill key functions in virus adaptation and proliferation in the host. They are also truly captivating proteins because of their functional versatility and conformational adaptability. Still, elucidating these proteins’ structures and conformational dynamics is a challenging task because they are small in size, highly dynamic, and heterogeneous in an oligomeric state (and possibly in tertiary structure too). Last but not least, they reside and function in diverse cellular membranes, imposing additional difficulties in assessing their structures. Thus, even in their oligomeric forms, these proteins are out of reach for the single-particle EM analyses and binding of antibodies or protein engineering is required to make these proteins amenable for EM studies.(Nygaard et al., 2020, Wentinck et al., 2022, Majeed et al., 2023) They are difficult (even impossible) to crystalize or truncated protein constructs and stabilizing mutations need to be introduced. Consequently, X-ray structures might not correspond to the most populated functional protein state.(Thomaston et al., 2015, Thomaston et al., 2019, Sanders et al., 2024) NMR can only access certain protein populations because of the requirements for high protein concentration in which an oligomer’s structure is typically modeled;(Ma, Marassi et al., 2002) ESR spectroscopy (CW and pulse [PDS spectroscopy]) can provide information about alteration in local protein dynamics, large-scale structural rearrangements upon transition between conformational states, and the oligomeric order of the protein, but high-resolution structural information is inaccessible with this method.(Jeschke, 2012, Georgieva et al., 2015, Georgieva, 2017, Sahu and Lorigan, 2020, Majeed et al., 2021) Molecular dynamics studies(Shukla et al., 2015, Kolokouris et al., 2021) and protein structure prediction using artificial intelligence-based, (e.g., AlphaFold; Fig. 3)(Jumper, Evans et al., 2021) can also help to characterize viroporins’ conformations, oligomeric states, and ion-conduction functions. Still, these proteins have unique amino acid sequences, imposing additional challenges on the homology-based predictions of their structures. Furthermore, AlphaFold has yet to gain the capacity to deal with highly dynamic and heterogeneous proteins in lipid bilayers.(Nussinov, Zhang et al., 2022).

Therefore, it is apparent that the combined effort of several structural and computational biology methods is needed to characterize in detail the multifaced structures of these small viral membrane proteins. In addition, overcoming the current hurdles to study the structures of these proteins will represent a significant methodological contribution to membrane’s structural biology.

## CRediT authorship contribution statement

**Arvin Saffarian Delkhosh:** Writing – original draft, Visualization, Investigation, Formal analysis, Data curation. **Elaheh Hadadianpour:** Writing – original draft, Visualization, Investigation, Formal analysis, Data curation. **Md Majharul Islam:** Formal analysis, Data curation. **Elka R. Georgieva:** Writing – review & editing, Writing – original draft, Visualization, Validation, Supervision, Resources, Project administration, Methodology, Investigation, Funding acquisition, Formal analysis, Data curation, Conceptualization.

## Declaration of competing interest

The authors declare the following financial interests/personal relationships which may be considered as potential competing interests: Elka R. Georgieva is a member of the Deputy Editorial Board of JSB and JSBX. She is the guest-editor of the special issue “Structural and computational biology in virus-host interactions”.

## Data Availability

Data will be made available on request.
