# Supplementary material for: Highly versatile small virus-encoded proteins in cellular membranes: A structural perspective on how proteins’ inherent conformational plasticity couples with host membranes’ properties to control cellular processes
Source: J Struct Biol X. 2024 Dec 11;11:100117. doi: 10.1016/j.yjsbx.2024.100117 (PMC11714672; doi:10.1016/j.yjsbx.2024.100117)
Supplement: Supplementary Data 1 [file mmc1.pdf]

SUPPLEMENTARY INFORMATION

Highly versatile small virus-encoded proteins in cellular membranes: A structural perspective on how proteins' inherent conformational plasticity couples with host membranes' properties to control cellular processes

Arvin Saffarian Delkhosh[#], Elaheh Hadadianpour[#], Md Majharul Islam, Elka R. Georgieva*

Department of Chemistry and Biochemistry, Texas Tech University, Lubbock, TX 79409

*Corresponding author, e-mail: elgeorgi@ttu.edu

[#]Equal contribution

EXPERIMENTAL PROCEDURES

Protein structure modeling

We used the artificial intelligence (AI) AlphaFold2 multimer software (Jumper, Evans et al. 2021) to predict the structures of HIV-1 Vpu pentamer, CoV E pentamer, HTLV-1 p13II monomer, HCV p7 hexamer, and P2B monomer. These structures are shown in Figure 3 in the main text of the manuscript. The AlphaFold program was run in ChimeraX. The structures were visualized using PyMOL software. To the best of our knowledge, these structural models were produced for this particular study and have not been published before.

Production, reconstitution in liposomes and nsEM of HTLV-1 p13II protein

The p13II protein was produced as a fusion construct with ubiquitin (p13II-Ub), as previously described (Georgieva, Borbat et al. 2020). The protein was reconstituted in DOPC/POPS (75:25 mol% of non-charged-to-charged lipids) at 1:300 protein-to-lipid molar ratio. The aliquot of 50 mM total lipid stock solution in 20 mM Tris/HCl pH 7.4, 150 mM NaCl, and 1 mM EDTA was taken and detergent Triton X-100 (10% stock solution in water) was added until the lipid bilayers were destabilized. The final Triton X-100 concentration was about 1-1.5 %.(Sanders, Borbat et al. 2024) Then calculated volume of p13II-Ub stock solution in a buffer of 20 mM Tris/HCl pH 7.4, 150 mM NaCl, and 100 uM TCEP was added to the destabilized lipid membranes and incubated for 1 h at 22 °C. Thereafter, the detergent was removed using BioBeads, which were changed several times, as previously described (Georgieva, Borbat et al. 2015). After the detergent was removed completely, proteoliposomes and empty liposomes were extruded 15 times using 200 nm polycarbonate membranes and a manual bench-top extruder (Avanti Polar Lipids). In parallel, protein-empty liposomes (with no p13II-Ub) were prepared using the same procedure but just buffer solution was added to the Triton X-100-detabilized lipid instead of adding p13II stock solution.

For negative staining EM (nsEM), we loaded 10μL of the proteoliposome and empty liposome samples onto a carbon-coated copper grid and allowed it to settle for 2 min 20 sec at room temperature. Then, we gently removed the solution on the grid using filter paper and then settled on the grid proteoliposomes and empty liposomes were stained by adding 10 μL of 1.6% 1uranyl acetate (UA) and incubated it for 2 min 20 sec. We again gently cleared the remaining UA solution using filter paper. We air-dried stained samples for 1-2 h at room temperature and then used them

for EM imaging. Afterward, we collected digital micrographs with a transmission electron microscope (TEM Hitachi H-7650), as described previously (Majeed, Adetuyi et al. 2023).

The nsEM images of empty liposomes and proteoliposomes are shown in Figure 3 D in the main text.

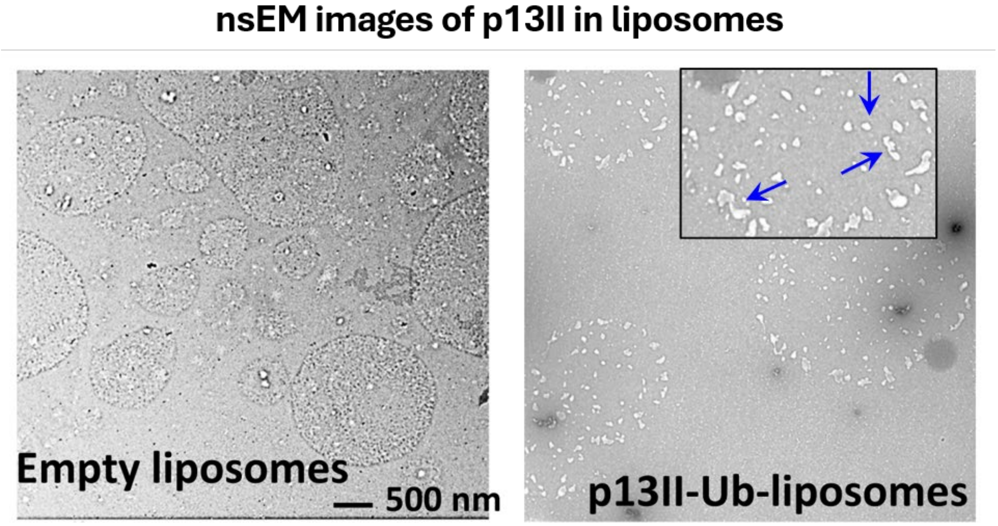

Supplementary Figure 1. nsEM of p13II reconstituted in liposomes. A representative image of empty liposomes is shown on the left, and liposomes containing p13II are shown on the right – large protein aggregates are visible. The samples were prepared as described in the EXPERIMENTAL PROCEDURES section. The image is the same as those in Figure 3D in the main text.

SUPPLEMENTARY REFERENCES

Georgieva, E. R., P. P. Borbat, C. Fanouraki and J. H. Freed (2020). "High-yield production in E. coli and characterization of full-length functional p13(II) protein from human T-cell leukemia virus type 1." Protein Expr Purif **173**: 105659.

Georgieva, E. R., P. P. Borbat, H. D. Norman and J. H. Freed (2015). "Mechanism of influenza A M2 transmembrane domain assembly in lipid membranes." Sci Rep **5**: 11757.

Jumper, J., R. Evans, A. Pritzel, T. Green, M. Figurnov, O. Ronneberger, K. Tunyasuvunakool, R. Bates, A. Zidek, A. Potapenko, A. Bridgland, C. Meyer, S. A. A. Kohl, A. J. Ballard, A. Cowie, B. Romera-Paredes, S. Nikolov, R. Jain, J. Adler, T. Back, S. Petersen, D. Reiman, E. Clancy, M. Zielinski, M. Steinegger, M. Pacholska, T. Berghammer, S. Bodenstein, D. Silver, O. Vinyals, A. W. Senior, K. Kavukcuoglu, P. Kohli and D. Hassabis (2021). "Highly accurate protein structure prediction with AlphaFold." Nature **596**(7873): 583-589.

Majeed, S., O. Adetuyi, P. P. Borbat, M. Majharul Islam, O. Ishola, B. Zhao and E. R. Georgieva (2023). "Insights into the oligomeric structure of the HIV-1 Vpu protein." J Struct Biol **215**(1): 107943.

Sanders, G., P. P. Borbat and E. R. Georgieva (2024). "Conformations of influenza A M2 protein in DOPC/DOPS and E. coli native lipids and proteins." Biophys J **123**(16): 2584-2593.
